# Pharmacokinetic and Pharmacodynamic Properties of Rosmarinic Acid in Rat Cholestatic Liver Injury

**DOI:** 10.3390/molecules23092287

**Published:** 2018-09-07

**Authors:** Jianbin Min, Hao Chen, Zipeng Gong, Xian Liu, Tian Wu, Weirong Li, Jiansong Fang, Tianlai Huang, Yingfeng Zhang, Wei Zhao, Chenchen Zhu, Qi Wang, Suiqing Mi, Ningsheng Wang

**Affiliations:** 1 Institute of Clinical Pharmacology, Guangzhou University of Chinese Medicine, Jichang Road 12, Guangzhou 510405, China; minjianbin@126.com (J.M.); oranous@126.com (X.L.); wtgoodluck924@yahoo.com (T.W.); liwr@gzucm.edu.cn (W.L.); fangjs@gzucm.edu.cn (J.F.); htl@gzucm.edu.cn (T.H.); zhucc@gzucm.edu.cn (C.Z.); wangqi@gzucm.edu.cn (Q.W.); misuiqing@gzucm.edu.cn (S.M.); nswang@gzucm.edu.cn (N.W.); 2 College of Food and Drug, Anhui Science and Technology of University, Fengyang 233100, Anhui, China; 15920554875@126.com; 3 Provincial Key Laboratory of Pharmaceutics, Guizhou Medical University, Beijing Road, Guiyang 550004, China; gzp4012607@126.com; 4 College of Chinese Medicine, Guangzhou University of Chinese Medicine, Jichang Road 12, Guangzhou 510405, China; zhangyingfeng@gzucm.edu.cn

**Keywords:** rosmarinic acid, cholestasis, pharmacodynamics, pharmacokinetics

## Abstract

The objective of this study was to evaluate the hepatoprotective and metabolic effects of rosmarinic acid (RA) in rats. RA [100 mg/kg body weight (BW)] was intragastrically (i.g.) administered to Sprague-Dawley (SD) rats once a day for seven consecutive days. The rats were then i.g. administered α-naphthylisothiocyanate (ANIT) (80 mg/kg once on the 5th day) to induce acute intrahepatic cholestasis after the last administration of RA. Blood samples were collected at different time points (0.083 h, 0.17 h, 0.33 h, 0.5 h, 0.75 h, 1 h, 1.5 h, 3 h, 4 h, 6 h, 8 h, 12 h, 20 h) after administration, and the levels of RA were estimated by HPLC. Plasma and bile biochemical analysis, bile flow rate, and liver histopathology were measured to evaluate the hepatoprotective effect of RA. The PK-PD curves showed obviously clockwise (AST and ALT) or anticlockwise (TBA, TBIL). Pretreatment with RA at different doses significantly restrained ANIT-induced pathological changes in bile rate, TBA, TBIL, ALT, AST (*p* < 0.05 or *p* < 0.01). The relationship between RA concentration and its hepatoprotective effects on acute cholestasis responses was assessed by PK-PD modeling.

## 1. Introduction

Cholestasis, a hallmark feature of hepatobiliary disease, is characterized by impairment of bile and bilirubin secretion [1,2,3] and alterations in the expression levels of hepatic membrane transporters and bile acid metabolic enzymes [2,4]. Cholestasis is often divided into two categories based on etiology, namely, intrahepatic and extrahepatic. Surgical removal of primary foci remains the main treatment for extrahepatic cholestasis, whereas the therapeutic options for intrahepatic cholestasis are limited because liver transplantation is only appropriate for certain patients. Thus, a better understanding of the molecular mechanisms underlying intrahepatic cholestasis is required to identify novel drug targets and improve current therapies.

**Figure 1:**
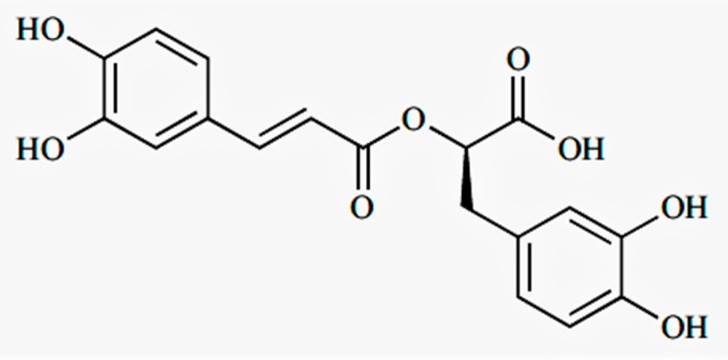
Chemical structure of rosmarinic acid (C_18_H_16_O_8_; 360.31).

Rosmarinic acid (RA) (Figure 1) is one of the most important and well-known polyphenolic antioxidants that is abundant in various medicinal plants of the Lamiaceae family and have historically been used in Traditional Chinese Medicine, which include *Rabdosia rubescens (Hemsl.) H. Hara.*, *R. amethystoides* (Benth.) Hara., *Rosmarinus officinalis* L., *Perilla frutescens* (L.) Britton, and *Salvia miltiorrhiza* Bunge [5,6,7]. *R. lophanthoides* is one of the major subspecies of *R. amethystoides* (Benth.) Hara., commonly known as *Xihuangcao* in China. It is a folk remedy that is commonly used for the prevention and treatment of hepatobiliary diseases in Southern China. Several studies have reported the role of RA and its pharmaceutical and biotechnological effects; for example, anti-colitic, antioxidant, anti-inflammatory, anti-leukemic, and anti-hepatic ischemia activities, as well as neuroprotective effects [8,9,10,11,12,13,14]. Its curative effects have been proven by both clinical applications and experimental research [7,15].

However, the pharmacokinetic (PK) and pharmacodynamic (PD) data about RA is limited and its anti-liver injury activity remains unclear. Upon oral administration, RA is rapidly absorbed (plasma values ca. 5 μmol/L) and metabolized into conjugated and/or methylated forms, which are mostly degraded and metabolized as conjugated forms of caffeic, ferulic, and *m*-coumaric acids [16]. 

To determine the pharmacological properties of RA, a more specific and specialized PK-PD model is essential. PK/PD integration, dose titration study, and PK-PD modeling are major methods that are employed to describe the dose-effect relationship of a drug by establishing the correlation between its PK and PD data. PK-PD modeling is a more effective approach to studies on PK/PD integration and dose adjustment. It has been widely used in pre-clinical and clinical studies and contribute to expressing drug efficacy over time and plasma concentration, thereby providing valuable references for optimizing the clinical dosage, improving the therapeutic efficacy, and reducing the toxic and side effects [17,18,19,20,21]. For example, PK/PD modeling was successfully applied for the selective dopamine D2 antagonist remoxipride, both in humans [22] and rats [23] to predict the pharmacological response beyond the tested conditions. Lv J reported that rhubarb is effective for the treatment of jaundice using the PK/PD model [24]. To date, no PK/PD model of RA in ANIT-induced cholestasis has been established to date. In our previous studies, we showed that RA is the predominant active component in a water extract of *R. serra*. Furthermore, we reported that *R. serra* imparts distinct hepatoprotective effects [25]. The present study assessed the pharmacodynamic and pharmacokinetic properties of RA in a cholestasis rat model induced by ANIT.

## 2. Results

### 2.1. HPLC Analysis

#### 2.1.1. HPLC Method Validation

**Figure 2:**
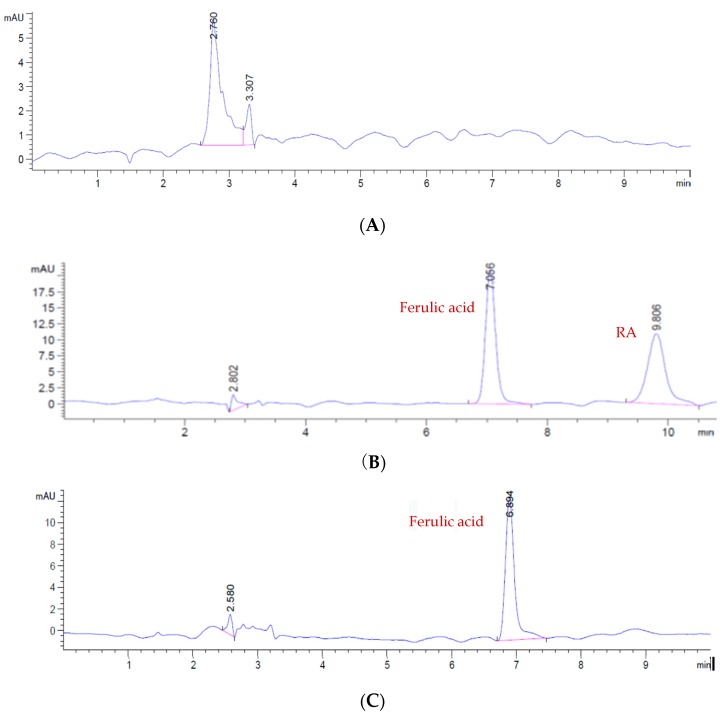
(**A**) HPLC–UV of blank (**B**) HPLC–UV of blank and ferulic acid. (**C**) HPLC–UV of blank control, ferulic acid, and RA.

The specificity was evaluated by comparing chromatograms of the blank plasma (from eight different rats) and blank plasma spiked with RA with plasma samples obtained from rats that received RA. Figure 2 shows that HPLC analysis of plasma samples showed no significant endogenous peaks that interfered with RA.

#### 2.1.2. Standard Curve

**Figure 3:**
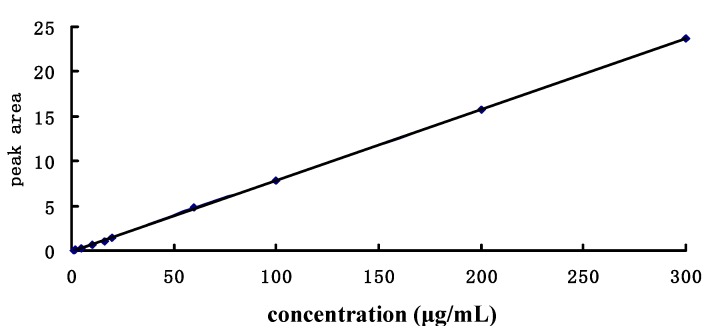
Standard curve of rosmarinic acid.

A standard curve was generated by plotting the analyte-to-internal standard peak area ratios (y) versus the concentration of the analyte standard (x) (Figure 3). Ten data points were used to generate the standard curve of plasma RA using linear regression. LOQ was defined as the lowest concentration on the standard curve with a signal-to-noise (S/N) ratio >10, and LOD was defined as the lowest concentration on the instrument with a signal–to–noise (S/N) ratio >3.

The resulting standard curve for RA was linear over the concentration range of 5–1500 μg/mL, the LOD was 0.22 μg/mL, and the LOQ was 0.74 μg/mL. The linear calibration curves equation was Y = 0.07955X − 0.1189, with a correlation coefficient r of 0.9999. These limits were deemed sufficient for our subsequent PK studies.

#### 2.1.3. Precision and Accuracy

The accuracy and precision of the PK analyses were evaluated by assessing the spiked serum samples at three concentration levels (35 μg/mL, 48 μg/mL, and 70 μg/mL), with each concentration consisting of five replicates. The deviation of the mean from the nominal value served as the measure of accuracy (set at ±15%). The intra-day precision determination of the five replicates was conducted on the same day. Inter-day precision was determined via repeating analysis for five consecutive days. Calibration curves of the same serum batch were generated each day for all determinations. Precision was expressed as the relative standard deviation (RSD%). The values for intra-day and inter-day precision and accuracy of the plasma samples are shown in Table 1. For each sample, the respective inter-day and intra-day RSDs and accuracy were all <5%. The results indicated that our method has an acceptable level of precision and accuracy.

#### 2.1.4. Stability

The stability of RA in rat serum was assessed using an auto-sampler for 6 h after storing at 4 °C for 6 h, frozen, and then thawed thrice, and long-term stability was evaluated after storage at −80 °C for seven days. Table 2 shows that the five plasma samples were stable in all these conditions.

#### 2.1.5. Extraction Recovery

The recovery and the absolute recovery of RA from rat serum samples were evaluated by comparing the resulting peak areas to those of the extracted blank samples that were spiked with the corresponding concentrations of the analytes after extraction (Table 3).

### 2.2. PK Research

**Figure 4:**
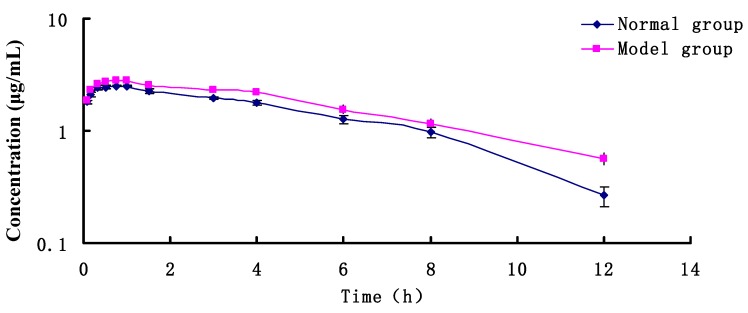
The T-C curve of RA in normal or cholestasis model rats.

After administration, the concentration-time relationship of RA in normal and rat model plasma samples were all fitted with the one-compartment model using the WinNonlin 6.4 software (Pharsight Corporation, Mountain View, CA, USA). The average plasma concentration-time curve is shown in Figure 4.

Figure 4 and Table 4 show that after administration, the RA plasma concentrations in the two groups all rapidly peaked (0.704 h and 0.988 h, respectively). In addition, the normal group reached a *T_max_* earlier than the model group. In addition, the normal control group showed an AUC_(0-∞)_ = 20.5 μg/L·h and CL = 4.876 L/h/kg, whereas the ANIT-induced cholestasis-model group exhibited an AUC_(0-∞)_ = 23.9384 μg/L·h and CL = 4.169 L/h/kg, indicating a significant increase in the AUC and a marked decrease in CL. *T_max_* and *C_max_* significantly decrease in the rat model, whereas that of the normal group did not change.

Taken together, the results indicated that the administration of RA resulted in a 15%, 40%, and 13% decrease in AUC, *T_max_*, and *C_max_*, respectively, whereas CL increased by 18%. These findings show an alteration in PK behavior of RA during cholestasis. In addition, bile secretion significantly decreased with cholestasis, thereby resulting in the accumulation of the drug.

### 2.3. PK-PD Modelling

**Figure 5:**
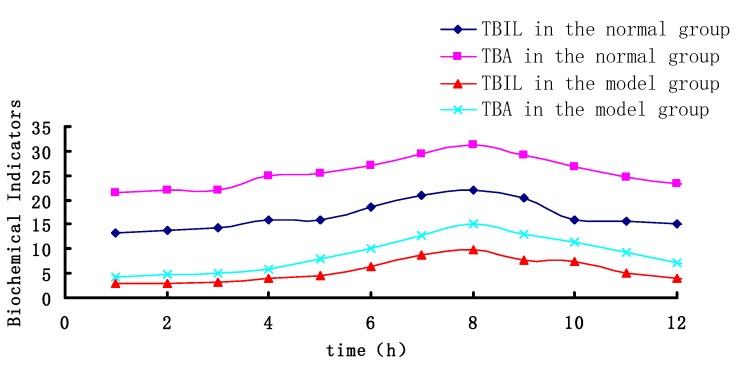
The time-effect correlation of RA in normal and cholestatic rats.

**Figure 6:**
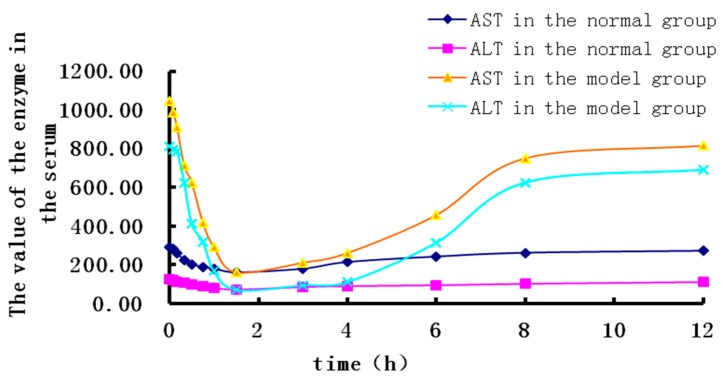
The time-effect correlation of RosA in normal and cholestatic rats.

Figure 5 and Figure 6 shows that the administration of RA results in a significant decrease in plasma AST and ALT level of the cholestatic rats 1.5 h after intragastric administration, as well as helps in the restoration of bile components (TBIL, TBA).

**Figure 7:**
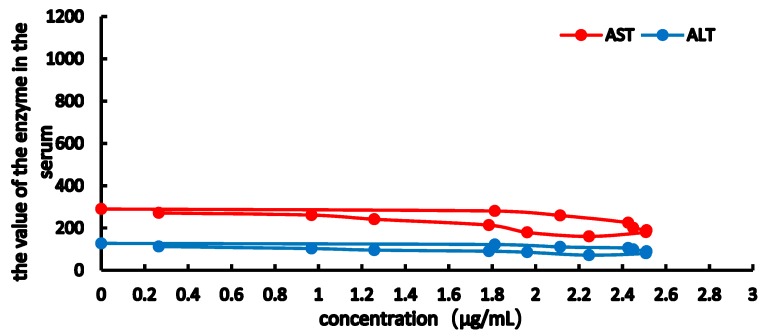
Concentration-effect correlation of RA in normal rats.

**Figure 8:**
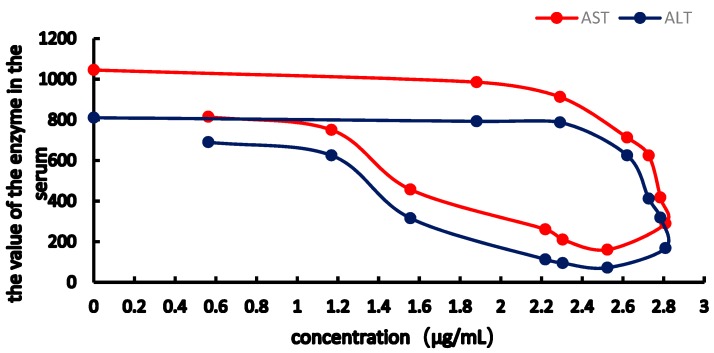
Concentration-effect correlation of RA in cholestatic rats.

**Figure 9:**
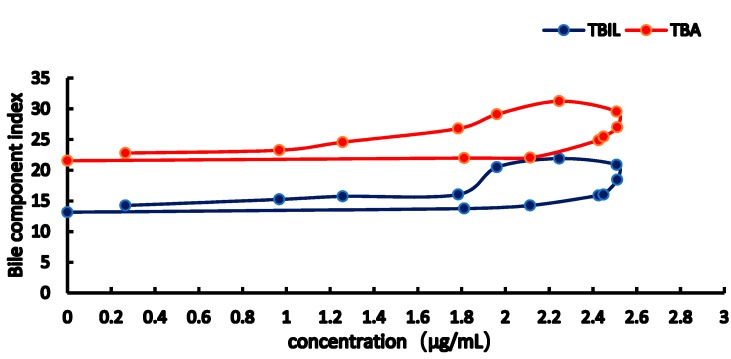
Concentration-effect correlation of RA in normal rats.

**Figure 10:**
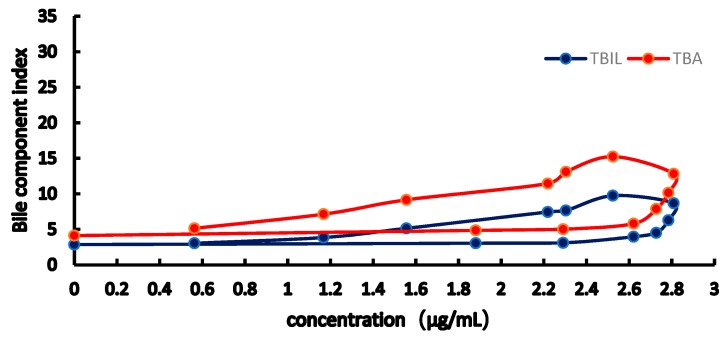
Concentration-effect correlation of RA in cholestatic rats.

From the Figure 7, Figure 8, Figure 9 and Figure 10, the results showed obviously clockwise (AST and ALT) or anticlockwise (TBA, TBIL). Pretreatment with RA at different doses significantly restrained ANIT-induced pathological changes in bile rate, TBA, TBIL, ALT, AST (*p* < 0.05 or *p* < 0.01). In Figure 7 and Figure 8, the clockwise tendency of AST and ALT means that the drug concentration lags behind the pharmacological effect. On the other hand, in Figure 9 and Figure 10, pharmacological effect lags behind the drug concentration make a counter-clockwise tendency. 

The PK and the PD data were analyzed using the WinNonlin 6.4 software and fitted to get the parameters of the PK-PD binding model.

From Table 5 and Table 6, The PK-PD model was established by combining the one-compartment PK model with the classical compartment-room model. According to the Sheiner effect room model, the effector chamber is a hypothetical chamber that is connected to the central chamber by a first-order kinetic process, which correlates the plasma concentration to the effect through the effector chamber.

The relationship between the two groups can be expressed as a Sigmoid-*E_max_* model:

(1) $$\;E=E_{0}+\frac{E_{max}\times C_{e}^{\gamma }}{EC_{50}^{\gamma }+C_{e}^{\gamma }}\;$$

For the inhibitory model, the above equation can be written as follows:

(2) $$\;E=E_{0}-\frac{I_{max}\times C_{e}^{\gamma }}{IC_{50}^{\gamma }+C_{e}^{\gamma }}\;$$

where *E*_0_ is the minimum drug concentration when drug concentration or drug dose is infinite, *EC*_50_ is the concentration of the drug in the effector room corresponding to 50% of the maximum effect, *E*_0_ is the drug concentration in the effector room, and γ is the Hill coefficient.

According to the above data, the content of TBIL and TBA in the cholestasis model can be respectively expressed as:

(3) $$E=2.855+\frac{20.279\times C_{e}^{2.895}}{3.410^{2.895}+C_{e}^{2.895}}\;\mathrm{and}$$

(4) $$\;E=4.183+\frac{46.279\times C_{e}^{2.122}}{4.589^{2.122}+C_{e}^{2.122}}\;$$

The concentration of AST and ALT in the cholestasis model of RA can be respectively expressed as follows:

(5) $$E=1175.756-\frac{6765.54\times C_{e}}{14.369+C_{e}}\;\mathrm{and}$$

(6) $$\;E=978.187-\frac{3660.634\times C_{e}}{7.460+C_{e}}\;$$

An indirect model with one compartment thus allowed the characterization of the PK-PD relationship of RA between the normal and model groups.

## 3. Discussion

RA is a natural water-soluble polyphenol that is often stored at low temperatures and in the dark. Previous studies have shown that RA and methylated RA can be detected in the plasma after administration. The absorption, distribution, metabolism, and excretion of RA in the body takes 8–18 h. This indicates that RA can be partially absorbed, metabolized, or biotransformed into other substances in rats.

In this study, we investigated the changes in RA in healthy and cholestasis model rats after a single oral administration. The study also assessed various extraction methods for plasma protein using ethyl acetate and methanol-water, methanol-phosphoric acid and methanol-acetic acid water on the peak of RA was studied. The results indicated that methanol-acetic acid water is the optimal mobile phase.

RA parameters were calculated using a PK software. The AUC_(0-∞)_ of RA (100 mg/kg) in normal control group was 17.29 mg·h/mL. The AUC_(0-∞)_ of RA in rats was increased by 23.11 mg·h/mL after ANIT-induced cholestasis, which may be due to liver injury and a significant decrease in bile secretion during cholestasis, thereby leading to drug accumulation and ultimately liver toxicity.

A PK model was established using WinNonlin 6.4 software. The PK-PD binding model was fitted with the PK-PD Link program module in WinNonlin 6.4. The PK model was determined using various model fitting methods. The experimental data were expressed as x ± s. The PK and PD parameters were compared between the two groups using the *t*-test, and differences with a *p* < 0.05 were considered statistically significant.

The TBIL, TBA of the E-C curve shows a counterclockwise ring, indicating that TBIL and TBA in bile significantly lagged behind the plasma concentration. The AST, ALT E-C curve showed a clockwise ring, showing that RA has maximum pharmacological activity, inhibiting the increase in AST, ALT levels, and at the same time, thereby delaying the pharmacological effects of RA. The levels of TBIL and TBA in bile and AST, ALT in serum were measured, and a significant improvement was observed 90 min after RA administration. In addition, the AUC of RA in the cholestasis rat model was higher than that in normal rats, which may be caused by bile acid excretion, thereby reducing RA elimination and half-life and increasing the maximum RA concentrations.

*E_max_* represents the largest drug effect, reflecting the intrinsic activity of RA, wherein the greater the value, te greater the intrinsic activity. *EC*_50_ is the concentration of drug in response to 50% of the maximal effect, which reflects the affinity of a drug to acceptor. The smaller the *EC*_50_, the greater the affinity of a drug to its receptor. *γ* reflects the size of the slope of the central section of the curve, which reflects the selectivity and sensitivity of a drug.

Based on the TBIL and TBA levels, the *E_max_* model group was 24% and 48% of that of the control group, indicating that RA has a certain recovery TBIL, TBA function. *EC*_50_ analysis showed that RA has a higher affinity for TBIL. The AST and ALT levels show that *I_max_* is the greatest benefit of inhibition, wherein the greater the value, the greater the intrinsic activity of a drug, indicating that RA-induced inhibition of AST, ALT increases is better. The *γ* values indicate that the RA concentration-effect curve is steeply S-shaped, suggesting that there is a need to further investigate the safety of using RA as a drug. After this relationship has been established, this model could provide useful information or explanation of the therapeutic action of RA in ANIT-induced acute cholestasis.

Bile is a unique and vital aqueous secretion of the liver that is formed by hepatocytes and is modified downstream by absorptive and secretory properties of the bile duct epithelium. Cholestasis is defined as the impairment of normal bile flow resulting either from a functional defect at the level of the hepatocytes or from obstruction at the bile duct level and might result from infection, drugs, and autoimmune, metabolic, or genetic disorders [26,27,28,29]. Bile is an alkaline body liquid produced by the liver cells of most vertebrates. After secretion, bile enters the gallbladder where it is concentrated or directly delivered to the intestinal lumen. Bile consists of ~95% water that contain various endogenous solid constituents such as bile salts, bilirubin phospholipids, cholesterol, amino acids, and steroids. Meanwhile, exogenous drugs, xenobiotics, and environmental toxins are also secreted through bile [30].

Interruption of bile flow leads to the accumulation of bile acids and other bile components in the liver and, ultimately, hepatobiliary toxicity. Liver injury is characterized by elevated serum bile acids and bilirubin, the increased aspartate aminotransferase activity, and histopathological lesions. TBA and TBIL increased when the secretion of bile is obstructed in cholestasis. A significant increase in ALT and AST levels are hallmarks of liver injury. Bsep and Mrp2 are critically involved in this process, and many kinds of bile acid transporters have been demonstrated to play vital roles in maintaining hepatic bile acid homeostasis in absorption and excretion [31,32].

ANIT is a hepatotoxicant that was used to simulate human intrahepatic cholestasis in rats. The liver intrahepatic cholestasis injury caused by ANIT is thought to reflect the potential toxicity of this chemical in hepatocytes and biliary cells [26,33]. ANIT damages bile duct epithelia, thereby inducing cholestasis. The ANIT-induced pathological changes in the liver tissues include hyperplasia of the bile duct epithelial cells, necrosis of liver cells, inflammatory cell infiltration, and hyperplasia of collagen fibers.

In our previous studies, three doses of ANIT (60 mg/kg, 65 mg/kg, and 100 mg/kg, respectively) were intragastric administered to rats, and bile flow rate and pathological changes were observed, indicating liver cholestasis injury, including edema, bile duct obstruction, serious interlobular duct epithelial apoptosis, or necrosis [25]. Based on liver pathologic grades, 65 mg/kg was determined to be suitable for our experiment. The ANIT dose 48 h before the last administration of RA was also determined.

## 4. Conclusions

The present study assessed the PK and PD properties of RA in a cholestasis rat model. The PK behavior of a single dose of RA that was administered orally was evaluated using a single-compartment model. The PK and PD parameters were also fitted.

## 5. Materials and Methods

### 5.1. Drugs and Reagents

RA (Chengdu Must Biological Technology Co., Ltd., Chengdu, China, batch number 1411507, purity 99.4%); ferulic acid (Chengdu Must Biological Technology Co., Ltd; batch number: 150305, purity ≥ 98%); bilirubin direct (DBIL), total bile acid (TBA), and total bilirubin (TBIL) reagent (BioSino Biological Technology Co., Ltd., Beijing, China; batch numbers 140621, 140721, and 140531, respectively); aspartate transaminase (AST) and alanine aminotransferase (ALT) (Shanghai Kehua Bio-Engineering Co., Ltd., Shanghai, China; batch numbers 20140214 and 20140521, respectively); ANIT (Sigma, San Jose, CA, USA; batch number STBC5577V); and ultrapure water, prepared by our laboratory, was used throughout of this research. All other chemicals were of analytical grade. The extraction procedure was as follows: approximately 2 kg of dried *R. lophanthoides* were crushed into small pieces and filtered through a 40-mesh (0.45-mm) sieve, then the pieces were soaked in 40 L of ethanol at room temperature for 1 h. RA was extracted from ethanol using the heating circumfluence method at 80 °C twice, each for 2 h. A macroporous resin X-5 was employed absorb the extract at a rate of 2 BV/h, which was then washed with 80% ethanol, and the eluent was concentrated by reducing pressure followed by drying by vacuum, yielding 39.32 g of RA.

### 5.2. Animals

SPF-grade male Sprague-Dawley rats, weighing 200 g–220 g, were purchased from the Medical Experimental Animal Center of Guangdong Province (Guangdong, China). The animals were housed under controlled standard conditions (25 ± 2 °C), relative humidity (60 ± 10%) with the natural light–dark cycle, and free access to standard rat food (laboratory rodent chow) and water for one week prior to the experiment. The animals were fasted overnight with free access to water prior to drug administration. All animal experiments were performed in accordance with the Guidelines for the Care and use of Laboratory Animals of Guangzhou University of Chinese Medicine.

### 5.3. Instruments

Clinical chemistry analysis of serum samples was conducted with an Automatic Biochemistry Analyzer (ECHO, Milan, Italy). Chromatographic analysis was performed using an Agilent 1100 series HPLC system (Agilent Technologies Inc., Santa Clara, CA, USA) consisting of a quaternary pump, a diode array detector (DAD), an autosampler, a vacuum degasser, and a column oven. Data analysis and model establishing were conducted using Winnolin 6.4 (Pharsight Corporation, Mountain View, USA).

### 5.4. Liquid Chromatography

RA was separated using a Luna C_18_ column (250 mm × 4.6 mm, Phenomenex, Torrance, CA, USA), and separation was performed on an Agilent 1100 series HPLC equipment. The mobile phase composition included methanol (A) and 1% acetic acid (B) (A:B = 45:55, *v*/*v*). The detection wavelength was 330 nm. Isocratic elution was performed at a flow rate of 1 mL/min for a total run time of 12 min and an injection volume of 10 μL. Column temperature was maintained at 30 °C.

### 5.5. Standard Solution and Sample Preparation

Stock solutions of RA (2 mg/mL) and ferulic acid [internal standard (IS), 1 mg/mL] in methanol were prepared. Working solutions of these analytes were freshly prepared by diluting the standard solutions in methanol to concentrations of 1500, 1000, 500, 300, 100, 90, 50, 25, 10 and 5 μg/mL. In addition, a working IS solution at a concentration of 140 μg/mL was prepared.

Corresponding standard (10 μL) and ethyl acetate (300 μL) was added into 100 μL blank serum, mixed together for 10 min then 10 μL of the internal standard (ferulic acid) and 10%HCL (10 μL) was put into the mixture in turn, mixed again for 5mins, and centrifuged (12,000 r/min) for 15 min. The substratum (aqueous layer) was extracted with ethyl acetate and merged with organic layer, and flushing with nitrogen, redissolved with methanol (50 μL) and injected with 10 μL. The standard curve concentration was: 300, 200, 100, 60, 20, 16, 10, 5, 2 and 1 μg/mL. All solutions that contained the IS were stored at 4 °C and were prepared for PK studies.

Each sample was mixed for 1 min and centrifuged at 12,000 rpm for 15 min at 4 °C. After stratification, the aqueous layer was separated and extracted twice with ethyl acetate. The ethyl acetate solution were dried under nitrogen and then dissolved in 100 μL methanol, mixed for 30 s, centrifuged (12,000 rpm) for 15 min, and then 10 μL was injected into the HPLC for analysis.

To validate our method, three concentrations of the standard solution containing RA (35 μg/mL, 48 μg/mL, 70 μg/mL) and IS were used for preparing QC plasma samples.

### 5.6. PK and PD Research

Sixteen Sprague-Dawley rats were randomly divided into two experimental groups: control group and model group. Animal models of intrahepatic cholestasis were established (80 mg·kg^−1^) 48 h treatment with ANIT, and RA (100 mg/kg) was orally administered before time zero. Blood samples were collected at 0.083, 0.17, 0.33, 0.5, 0.75, 1, 1.5, 3, 4, 6, 8, 12 and 20 h after oral administration. Orbital venous blood samples were collected into heparinized Eppendorf tubes and left to stand for 1 h. Then, the blood samples were then centrifuged at 3800 rpm for 10 min at 4 °C to isolate the serum, which were transferred into clean tubes and stored at −80 °C until analysis.

Before the operation, all rats were fasted and supplied with water for 8 h. Approximately 48 h after administration of ANIT, the rats were anesthetized and operated by bile duct cannulation on the 3rd day. The right side common carotid artery was separated for intubation. Then, RA (100 mg/kg) was given to the animals, and 0.1 mL blood samples and 0.25 mL bile were in turn collected at 0, 0.083, 0.167, 0.33, 0.5, 0.75, 1, 1.5, 3, 4, 6, 8 and 12 h, respectively. Bile from each rat was collected for 5 min, followed by isolation of the liver. The blood samples were allowed to coagulate for 10 min to obtain serum, followed by centrifugation at 3000 rpm for 10 min. All blood and bile samples were stored at 4 °C until analysis.

### 5.7. Blood Biochemical Determinations

After bile collection, the animals were sacrificed. Blood samples were stored in Eppendoff tubes and used in chemical analysis, including ALT and AST. TBIL and TBA in bile were also assayed using kits. Analysis was conducted using an automatic biochemical analyzer (Italy Echo instruments, Milan, Italy).

### 5.8. Analysis

The PK data were analyzed using the WinNonlin 6.4 software which was provided by Dr. Zipeng Gong. Oral administration of RA was performed to determine blood PK parameters, including elimination half-life (t_1/2_), mean retention time of drug (MRT), area under the concentration-time curve (AUC_(0-t)_ and AUC_(0-∞)_), clearance (CL), and the apparent volume of distribution (V_d_).

Analysis was performed using the t-test and one-way ANOVA with test using SPSS 21.0 (IBM company, Chicago, IL, USA, and differences with a *p* < 0.05 were considered statistically significant and those with a *p* < 0.01 were regarded as highly significant.

## Figures and Tables

**Table 1:** The precision of the RA HPLC method using rat plasma samples (*n* = 5).

Concentration/µg·mL^−1^	RSD (%)		
	Inter-Day Precision	Intra-Day Precision	Accuracy
35	1.44	3.84	2.89
48	2.34	4.65	2.58
70	1.44	3.85	1.37

**Table 2:** The stability rate of RA on the plasma in rats (*n* = 5).

Concentration/µg·mL^−1^	RSD%		
	6 h at 25 °C	6 h at 4 °C	7 d at −80 °C
35	1.44	2.05	5.72
48	2.34	2.86	5.98
70	1.44	2.32	5.31

**Table 3:** The recovery rate of RA in rats plasma (x ± S, *n* = 5).

Concentration/μg·mL^−1^	The Recovery	RSD/%	The Absolute Recovery	RSD/%
35	96.7 ± 3.72	3.84	91.48 ± 1.00	1.09
48	94.54 ± 1.61	1.71	95.82 ± 1.71	1.78
70	89.98 ± 1.92	2.14	88.97 ± 2.80	3.15

**Table 4:** Pharmacokinetic parameters of RA in normal and cholestasis model rats (x ± S, *n* = 8).

Pharmacokinetic Parameters	Unit	Normal Group	Model Group
AUC	mg·h/mL	20.500 ± 1.199 **	23.984 ± 0.678
K_01_HL_	h	0.181 ± 0.061 **	0.303 ± 0.092
K_10_HL_	h	4.930 ± 0.419	4.799 ± 0.264
CL__F_	L/h/kg	4.876 ± 0.285 **	4.169 ± 0.118
*T_max_*	h	0.704 ± 0.120	0.988 ± 0.153
*C_max_*	mg/mL	2.542 ± 0.054 **	2.876 ± 0.082
V__F_	L/kg	34.683 ± 1.433	28.867 ± 1.438
K_01_	h^−1^	3.827 ± 1.283	2.291 ± 0.698
K_10_	h^−1^	0.141 ± 0.012	0.144 ± 0.008

Note: Compared to the model control group ** *p* < 0.01.

**Table 5:** Parameters of the model and blank groups.

Parameter	Model Group		Blank Group	
	AST	ALT	AST	ALT
*I_max_*	6765.54	3660.634	855.64	338.667
*IC*_50_	14.369	7.460	12.640	12.702
*E*_0_	1175.756	978.187	309.569	129.138
K_e0_	1.476	1.346	2.538	1.662

**Table 6:** Parameters of the model and blank groups.

Parameter	Model Group		Blank Group	
	TBIL	TBA	TBIL	TBA
*E_max_*	20.279	46.028	84.572	96.36
*EC*_50_	3.410	4.589	4.769	6.235
*E*_0_	2.855	4.183	13.935	21.85
Gamma	2.895	2.122	3.1445	2.283
K_e__0_	1.780	1.535	2.067	1.918
